# Influence of *NUCB*/Nesfatin-1 Polymorphism on Treatment Response to Naltrexone/Bupropion SR in Binge Eating Disorder and Obesity

**DOI:** 10.3390/biomedicines12020451

**Published:** 2024-02-17

**Authors:** Elvira Anna Carbone, Mariarita Caroleo, Marianna Rania, Renato de Filippis, Francesca Condoleo, Federica Catalano, Matteo Aloi, Pasquale De Fazio, Franco Arturi, Marta Letizia Hribal, Teresa Vanessa Fiorentino, Cristina Segura-Garcia

**Affiliations:** 1 Department of Medical and Surgical Sciences, University Magna Graecia of Catanzaro, 88100 Catanzaro, Italy; elvira.carbone@unicz.it (E.A.C.); francesca.condoleo92@gmail.com (F.C.); arturi@unicz.it (F.A.); hribal@unicz.it (M.L.H.); vanessa.fiorentino@unicz.it (T.V.F.); 2 Department of Health Sciences, University Magna Graecia of Catanzaro, 88100 Catanzaro, Italymatteo.aloi@unicz.it (M.A.); defazio@unicz.it (P.D.F.); 3 Center for Clinical Research and Treatment of Eating Disorders, University Hospital Renato Dulbecco, 88100 Catanzaro, Italy; marianna.rania@hotmail.it; 4 Department of Clinical and Experimental Medicine, University of Messina, 98125 Messina, Italy

**Keywords:** *NUCB2*, polymorphism, nesfatin-1, binge eating disorder, obesity, food addiction, eating behaviors

## Abstract

*Background and Objectives*: The *NUCB2* gene and its polymorphisms were identified as novel players in the regulation of food intake, potentially leading to obesity (OBE) and altered eating behaviors. Naltrexone/bupropion SR (NB) showed good efficacy and tolerability for treating OBE and altered eating behaviors associated with binge eating disorder (BED). This prospective study investigates the influence of *NUCB2* gene polymorphism on NB treatment response in OBE and BED. *Materials and Methods*: Body mass index (BMI), eating (EDE-Q, BES, NEQ, GQ, Y-FAS 2.0) and general psychopathology (BDI, STAI-S) were evaluated at baseline (t0) and after 16 weeks (t1) of NB treatment in patients with OBE and BED (Group 1; *N* = 22) vs. patients with OBE without BED (Group 2; *N* = 20). Differences were evaluated according to the rs757081 *NUCB2* gene polymorphism. *Results*: *NUCB2* polymorphism was equally distributed between groups. Although weight at t0 was higher in Group 1, weight loss was similar at t1 in both groups. BMI was not influenced by *NUCB2* polymorphism. In Group 1, the CG-genotype reported significant improvement in eating psychopathology while the GG-genotype reported improvement only for FA. No differences were observed in Group 2. *Conclusions*: Patients diagnosed with BED and treated with NB exhibited a more favorable treatment response within the CG-genotype of the *NUCB2* polymorphism.

## 1. Introduction

Central food intake is mainly regulated by two systems: the hypothalamic circuit of melanocortin (i.e., the regulator of appetite and food intake) [1], and the mesolimbic dopamine circuit (i.e., the reward pathway) [2]. The first one is related to the “homeostatic control of feeding”, in which food intake seems to be limited to satisfy an individual’s biological needs and regulate energy balance; the second one is related to the “non-homeostatic” feeding or “hedonic feeding” and refers to eating for pleasure or the reward associated with food intake when over-consuming palatable food [2]. Interestingly, the systems involved in homeostatic and non-homeostatic feeding are not entirely separated, as multiple connections between these two systems exist [3]. Different central and peripheral stimuli may interact with these systems to control the food intake [4].

Recently, nesfatin-1 and its *NUCB2* gene have been identified as novel players in the pathways involved in the homeostatic and hedonic regulation of food intake [5,6]. The secreted protein nesfatin-1 has not only peripheral (i.e., on body temperature, energy expenditure, glycemic control) [7,8,9] but also central actions (i.e., on the reward circuitries to modulate hedonic feeding-related behaviors) [10,11] crossing the blood–brain barrier [12] or directly expressed in the reward-related brain regions (i.e., ventral and dorsal striatum, lateral hypothalamus, dorsal and dorsolateral tegmental nucleus) [13].

As noted, dysregulated eating behaviors are a primary factor that contributes to the increased rate of obesity worldwide [14] and genetic studies are growing to elucidate the possible link between the *NUCB2*/nesfatin-1 gene and the role of single nucleotide polymorphisms in the development of obesity (OBE) [15]. Recently, *NUCB2* gene polymorphisms (e.g., rs1330, rs214101 and rs757081) have been associated with OBE [16], suggesting that *NUCB2* might be involved in the regulation of energy homeostasis and food intake [15]. Evidence has emerged in particular for the c.1012C>G polymorphism (Q338E or rs757081), supporting the connection between the rs757081 variant and higher BMI [16], fat free mass [16], and childhood adiposity [15,17].

On the other hand, obesity is common among patients with binge eating disorder (BED) [18], leading to a considerable medical burden due to the metabolic complications and the decrease in quality of life [19]; novel research is trying to disentangle the genetic contribution of the *NUCB2*/nesfatin-1 gene and the co-occurrent BED [20].

Hypothalamic mRNA levels of the *NUCB2* product seem to be sensitive to fasting [21]. Indeed, a pre-clinical study demonstrated that increased levels of both mRNA [21] and nesfatin-1 in the hypothalamus regulate feeding behavior by stimulating the synthesis of pro-opiomelanocortin (POMC). This evidence supports not only the stimulatory role for nesfatin-1 on POMC synthesis [22] through the melanocortin system and its melanocortin receptors (MCR) (i.e., MC3/4R) [23] but also the action on the dopaminergic reward pathway to inhibit food intake [24]. In addition, recent studies suggested that the secreted protein nesfatin-1 regulates anxiety and depressive symptoms [25,26].

Based on this evidence, it may be hypothesized that the polymorphism of *NUCB2* could influence *NUCB2* gene function participating in nesfatin-1 expression and in the regulation of the food intake system leading not only to an increased BMI but also increasing dysfunctional eating behaviors [20].

Among the new available treatment for treating OBE and altered eating behaviors, naltrexone/bupropion SR (NB) has shown good efficacy and tolerability in the treatment of OBE [27,28,29] and a previous study demonstrated that it was effective in reducing altered eating behaviors, in particular food addiction, in high-weight patients with BED [30] due to its action both on the hypothalamus, stimulating and increasing POMC activation [28], and on the modulation of the reward circuits [31].

Because of the involvement of the *NUCB2* gene in the melanocortin pathway that regulates energy homeostasis and the reward system, and the synergic effect of NB, we assume that polymorphism of *NUCB2*/nesfatin-1 could influence the treatment response to NB. Thus, this study aims at investigating the influence of rs757081 *NUCB2* gene polymorphism in NB treatment response by evaluating changes in BMI, restraint, eating behaviours, depression, and anxiety in patients with OBE and BED.

## 2. Materials and Methods

### 2.1. Participants

In this prospective clinical study, participants were consecutively recruited at the outpatient clinic for eating disorders of the University Hospital “Renato Dulbecco” of Catanzaro (Italy) from June 2021 to June 2023. Individuals were considered eligible if they reported the following: (1) a body mass index (BMI) ≥ 30 kg/m^2^; (2) an age ≥18 years and <65; (3) ability to answer self-report questionnaires; and (4) ability to give valid informed consent to the study. On the other hand, patients were deemed not eligible to be enrolled if they met the following criteria: (1) diagnosis of schizophrenia spectrum disorders or bipolar disorder according to DSM-5 criteria; (2) alcohol or substance abuse disorder in the previous 6 months; (3) pregnancy, or childbirth and/or breastfeeding; (4) severe and documented comorbid medical conditions (i.e., diabetes mellitus, uncontrolled hypertension, cancer disease, seizure disorders); (5) antidiabetic or hypoglycemic therapy, hormonal and pharmacological treatment potentially able to induce either cognitive impairment or metabolic changes and contraindications to the use of NB (e.g., severe kidney or hepatic failure); (6) absence of a credible, documented medical history.

Participants were duly informed individually about the aim, procedures, and anonymity of the study to obtain written informed consent before any procedures took place. The study protocol followed the ethical principles set out in the revised version of the Declaration of Helsinki [32] and was approved by the Ethical Committee of “Regione Calabria, sezione Area Centro” (identifier: 162/22.04.2021).

### 2.2. Procedures and Assessment

Data on physical health, anthropometric data (i.e., height, weight, and BMI), socio-demographics and documented medical comorbidities (e.g., diabetes, uncontrolled hypertension, cancer disease, seizure disorders) were collected for each participant with an ad hoc schedule. Clinical interviews were performed by trained psychiatrists (45 min alike) at first registration. The diagnosis of BED was confirmed or excluded during clinical interviews by a trained psychiatrist with expertise in the field of eating disorders through the Structured Clinical Interview for the DSM-5 (SCID-5-CV) [33]. Patients were evaluated at baseline (t0) and after 16 weeks of treatment (t1).

### 2.3. General and Eating Psychopathological Measures

All enrolled participants completed the following questionnaires:

- Eating Disorder Examination Questionnaire (EDE-Q 6.0) [34] to evaluate the psychopathology and symptomatology of eating disorders; we consider the Restraint subscale of EDE-Q as a measure of the reduction in food intake;
- Beck Depression Inventory (BDI-II) to assess depression severity [35];
- State–Trait Anxiety Inventory (STAI-S) to assess state anxiety [36];
- Binge Eating Scale (BES) [37] to measure the severity of behaviours, feelings and cognitions associated with binge eating;
- Night eating questionnaire (NEQ) [38], Italian version, to measure the night eating symptoms;
- Grazing Questionnaire (GQ) [39], Italian version, to evaluate the grazing behaviour;
- Yale Food Addiction Scale 2.0 (Y-FAS 2.0) [40], Italian version, to assess addiction-like eating behavior over the past 12 months; for this study, the total criteria were used.

The same assessment was administered to each participant after 16 weeks of treatment.

### 2.4. Patient Genotyping Analysis

Following similar strategies employed by other similar studies [16,20], a blood sample from patients was used for the genotyping of rs757081 *NUCB2*/nesfatin-1. Genomic DNA was extracted from a 200 mL peripheral whole blood sample by a Commercial DNA isolation kit (Promega, Madison, WI and Roche, Mannheim, Germany). DNA samples were stored at −20 °C until use. The real-time PCR systems were used for analysing the *NUCB2* gene polymorphism (rs757081). The cycling conditions for *NUCB2* were initial polymerase activation at 95 °C for 5 min, followed by 40 cycles with at 95 °C for 15 s and annealing/extension at 60 °C for 30 s. The TaqMan allelic discrimination assay (Assay ID# C_2261417_10 for *NUCB2*/nesfatin-1, Applied Biosystems, Foster City, CA, USA) was used to determinate the rs757081 *NUCB2*/nesfatin-1 genotype calls. Template DNA was amplified, and fluorescence was detected on a BioRad CFX-96 Thermal Cycler (Bio-Rad Laboratories, Inc., Hercules, CA, USA). All samples were analysed in duplicate.

### 2.5. Treatment Program

Participants initiated treatment with NB, taking one tablet per day, which contained 8 mg of naltrexone·HCl and 90 mg of bupropion·HCl. The dosage was gradually increased, reaching a maximum of two tablets twice a day by the fourth week. If patients experienced manageable side effects such as constipation, tinnitus, or nausea, the therapy dose was reduced to the minimum level needed for effectiveness. Patients were advised to maintain their usual eating and daily routines, except for engaging in moderate aerobic physical activity. Additionally, behavioural counselling to promote a healthy lifestyle was provided to each participant throughout the entire treatment duration. Adherence to treatment was evaluated and a weekly side-effects checklist was administered by a research clinician. During each visit, medication refills were accompanied by a review of medication compliance and dosing schedules. Pill bottles were returned for pill counts post-treatment.

### 2.6. Data Analysis

Descriptive statistic analysis (frequencies and percentages for categorical variables, means and standard deviations for continuous variables) were used to analyze the socio-demographic and clinical variables and depict the main results of tests. Differences between groups were examined through independent sample *t*-tests and chi- squared tests with Yates correction, as appropriate. The General Linear Model (GLM) with repeated measures was used to evaluate changes in BMI, restraint, grazing, binge eating, nigh eating, food addiction, depression, and anxiety after treatment with NB. For significant results, eta-squared (η^2^) values for GLM, and Cohen’s d for Student’s *t*-test, were calculated as measures of effect size. The level of statistical significance was set at *p* ≤ 0.05. The data analysis was performed with the Social Sciences Statistical Package, Version 26.0 (SPSS Inc., Chicago, IL, USA).

## 3. Results

### 3.1. Sample Description

Overall, 49 out of 58 participants initially invited for participation were considered eligible and enrolled in this study (5 did not fulfill the inclusion criteria, 2 met at least one exclusion criteria and 2 did not consent to give a blood sample). According to the diagnosis, patients were allocated to Group 1 (Ob-BED: high-weight patients with BED) or Group 2 (Ob-noBED: high-weight patients without BED).

In total, 42 out of 49 participants completed the 16-week treatment program, including 22 patients from Group 1 and 20 patients from Group 2. Only seven patients dropped out: three patients due to side effects (i.e., nausea, constipation) within the first four weeks, and four for not completing tests at follow-up.

Table 1 displays the socio-demographics of the final sample. The groups only differed in BMI (*p* = 0.012) and sex (*p* = 0.013). The rs757081 *NUCB2* polymorphism was equally distributed between groups (χ² = 2.637; *p* = 0.680). The distribution of alleles in the two groups was consistent with the Hardy–Weinberg equilibrium and comparable to other European populations, confirming a normal distribution.

### 3.2. Treatment Response

All the included patients demonstrated adherence to the treatment. Although weight at t0 was higher in the Ob-noBED group, a significant reduction in BMI was observed after treatment with NB (F = 121,453; *p* < 0.001, η^2^ = 0.786) in the whole sample. No effect of diagnosis (F = 1.268; *p* = 0.268) on weight reduction was evident as weight loss was similar in both groups of patients (Δ%BMI: Ob-BED = 7.9 ± 3.4 vs. Ob-noBED = 7.9 ± 3.6; F = 0.000; *p* = 0.993).

**Figure 1:**
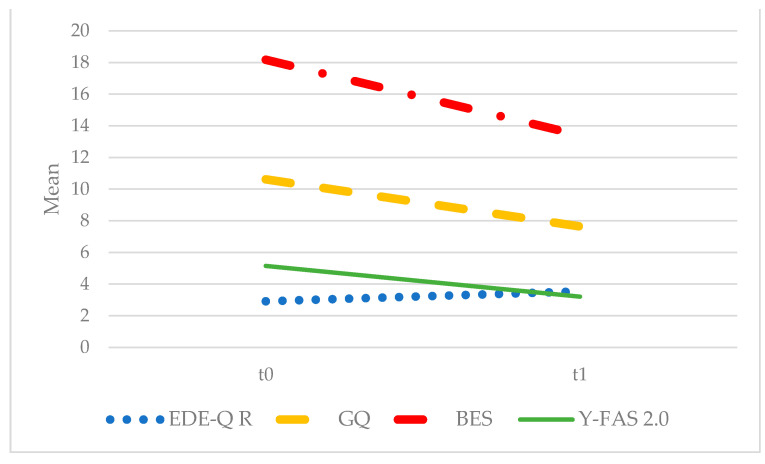
Significant differences after treatment with NB in the whole sample. BES: *Binge Eating Scale*; EDE-Q R: *Eating Disorder Examination Questionnaire-Restraint*; GQ: *Grazing Questionnaire*; Y-FAS 2.0: *Yale Food Addiction Scale 2.0*.

**Figure 2:**
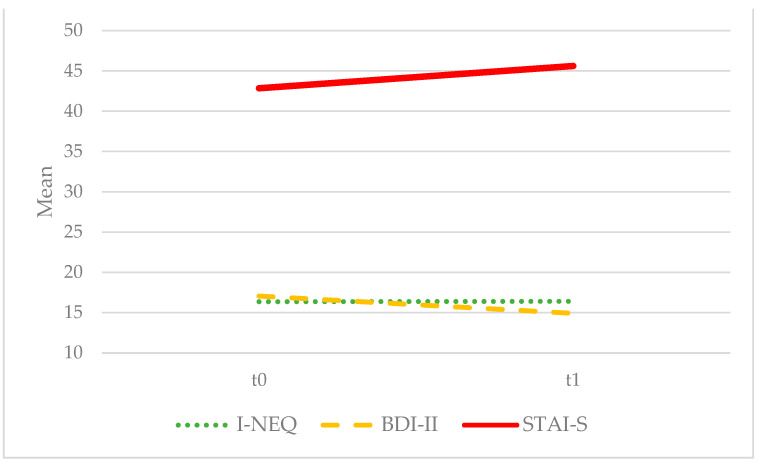
No significant differences after treatment with NB in the whole sample. BDI-II: Beck Depression Inventory II; I-NEQ: *Night Eating Questionnaire*; STAI-S: *State Trait Anxiety Inventory-State*.

The following significant differences were evident after treatment with NB (Figure 1): an increase in restraint (EDE-Q restraint: F = 4.398; *p* = 0.044; η^2^ = 0.124) and decrease in grazing (GQ total score: F = 5.997; *p* = 0.020; η^2^ = 0.154), binge eating (BES: F = 10.084; *p* = 0.003; η^2^ = 0.234), and food addiction (YFAS 2.0 criteria: F = 9.274; *p* = 0.005; η^2^ = 0.219). Conversely, night eating (I-NEQ total score: F = 0.230; *p* = 0.638), depression (BDI-II: F = 1.616; *p* = 0.213) and anxiety (STAI-S: F = 0.604; *p* = 0.443) scores did not show significant changes. No effect of diagnosis was observed for any of these variables (Figure 2): EDE-Q restraint: F = 1.541; *p* = 0.224; GQ: F = 0.274; *p* = 0.604; BES: F = 1.281; p = 0.266; YFAS 2.0: F = 0.763; *p* = 0.389; I-NEQ, F = 2.953; *p* = 0.104; BDI-II, F = 0.078; *p* = 0.782; STAI-S, F = 0.017; *p* = 0.898.

### 3.3. Influence of rs757081 NUCB2 Polymorphism on Treatment Response

**Figure 3:**
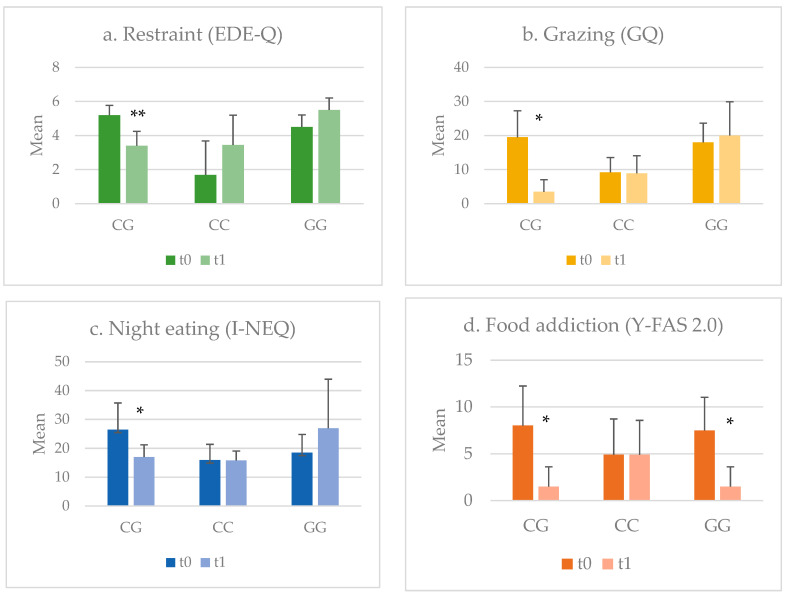
Changes in pathological eating behaviors according to rs757081 *NUCB2* polymorphism in Ob-BED group. EDE-Q: *Eating Disorder Examination Questionnaire*; GQ: *Grazing Questionnaire*; I-NEQ: *Night Eating Questionnaire*; Y-FAS 2.0: *Yale Food Addiction Scale 2.0*. *: *p* < 0.05; **: *p* < 0.001.

The main effect of *NUCB2* polymorphism on restraint (F = 15.448; *p* = 0.001; η^2^ = 0.775), grazing (F = 5.601; *p* = 0.021 η^2^ = 0.505), night eating (F = 3.932; *p* = 0.05 η^2^ = 0.417) and food addiction (F = 4.442; *p* = 0.039; η^2^ = 0.447) was evident in the Ob-BED group. Patients with the CG genotype reported statistically significant improvements in grazing, night-eating behaviors and food addiction even if their restraint decreased. On the other hand, the patients with the CC homozygosis genotype reported no significant changes in grazing, night eating and food addiction but only increased restraint. Increased grazing and night eating were reported among the GG genotype group even if restraint increased. Surprisingly, the improvement of food addiction (*p* = 0.039, F = 4.442, η^2^= 0.447) was also associated with the GG genotype (See Figure 3a–d). There was not a significant effect of *NUCB2* polymorphism on BMI (F = 0.658; *p* = 0.537; η^2^ = 0.107), BES (F = 1.975; *p* = 0.185; η2 = 0.264), BDI-II (F = 3.454; *p* = 0.069; η^2^ = 0.386) or STAI-S (F = 0.086; *p* = 0.918; η^2^ = 0.015).

No statistically significant effect of *NUCB2* polymorphism on treatment response was observed among the patients of the Ob-noBED group.

## 4. Discussion

To the best of our knowledge, this is the first study that investigated the influence of the *NUCB2*/nesfatin-1 gene polymorphism on NB treatment response in high-weight patients with and without BED. In our sample, both groups showed similar weight reductions and changes in general and eating psychopathology after treatment with NB. The results are in line with recent literature demonstrating that NB is a useful and safe treatment option [27,28] not only for weight loss [27,28] but also for improving pathological eating behaviors in high-weight patients with BED [30] due to its peculiar mechanism of action in controlling both hunger and appetite [41,42]. In our previous study [30], patients with BED exhibited better control of eating behaviors after treatment with NB by increasing food restriction and reducing binge eating, grazing and severity of food addiction.

The results of this preliminary study support the hypothesis that polymorphisms of *NUCB2*/nesfatin-1 influence the treatment response to NB. The rs757081 polymorphism is one of the most extensively studied SNPs in the *NUCB* gene. This SNP has two alleles, C and G, with the C allele being the ancestor. The genotype and rs757081allelic frequency in our sample are superimposable on the general population and this allows us to generalize our results. To date, previous studies have associated the rs757081 polymorphism of the *NUCB2* gene in adulthood (OR = 1.42) [16] and childhood (OR = 1.69) [17,20] with an increased risk of weight gain, higher metabolic rate, and impaired nesfatin-1 levels [15,16,17,43]. In particular, studies have shown that the C allele in overweight individuals may be a risk factor for metabolic syndrome [43] and elevated nesfatin-1 levels [20]. Conversely, the G allele is rarely found in overweight populations [17] and is considered protective against adiposity/weight gain [17]. Thus, it can be speculated that the C allele confers risk, and the G protects.

*NUCB2* is a relevant gene in determining eating behaviors, and its product nesfatin-1 is expressed in several brain regions implicated in the homeostatic and hedonic control of feeding and energy balance [5,10,24,44], supporting the demand for more studies on this gene, its polymorphisms and the nesfatin-1. Patients in our study, regardless of the diagnosis, exhibited weight loss and changes in eating behaviors after treatment, but a significant effect of *NUCB2* polymorphism was only evident in the Ob-BED group.

rs757081 *NUCB2* polymorphism could influence the *NUCB2* gene function; in line with the study by Caroleo et al. [20], patients with BED and the heterozygosis CG genotype may have an altered nesfatin-1 expression and lower plasma levels. This might result in decreased POMC stimulation with altered energy homeostasis and reward system regulation, and a consequent increase in food intake, altered eating behaviors and higher BMI. Nevertheless, our findings suggest that individuals with the heterozygosis CG genotype experience greater improvement of their eating behaviors following NB therapy than the homozygosis genotype. This suggests that NB might be more effective in individuals with the CG genotype compared to those with CC/GG genotypes that could have more altered *NUCB2* product levels. This enhanced response could be attributed to NB’s impact not only on the melanocortin system but also on the reward system. *NUCB2* is expressed in hypothalamic areas [5] and in the reward-related brain regions [10]. *NUCB2* can modulate the release of nesfatin-1 and other neurotransmitters [10,45], and, consequently, food intake and eating behavior. NB seems able to directly modulate the POMC and the dopaminergic systems regulating food intake and food reward in the presence of the heterozygosis CG *NUCB2* polymorphism and may be not useful in homozygosis genotypes.

The reduction in EDE-Q restraint in the CG genotype is likely to be considered consequential to an overall psychopathological improvement observed in patients.

Additionally, to the best of our knowledge, no previous study reported on the association between rs757081 *NUCB2* and FA reduction in patients treated with NB. We observed a significant influence of the CG and GG genotypes on NB-related FA reduction in the Ob-BED group. The authors’ opinion is that NB was able to increase dopamine in the mesolimbic system, thus re-establishing a partial balance and reducing FA. The results may suggest that patients with FA alone might benefit from NB treatment not only if they carry the CG genotype but also the GG genotype and this may also suggest that FA is a different and unique construct that deserves further in-depth investigation.

We did not observe any significant influence of rs757081 *NUCB2* on BMI and affective symptoms. The missing significance may be explained by the limited sample size and the limited follow-up. Therefore, further studies with a larger sample size and longer follow-up on *NUCB2* are warranted.

The limitations of this preliminary report also need to be acknowledged. First, the use of a relatively small sample size to generalize genetic findings and the absence of a randomization or placebo-controlled study design should be considered. Second, the use of self-report questionnaires, although well-validated tools, may have been affected by recalling bias leading to the underestimation of eating behaviors or affective symptoms. Third, this study only evaluated the effect of one polymorphism, and clarifying whether this gene has a direct relationship with eating behavior is not possible. Forth, the range in the proportion of men to women may have impacted our results. Yet, this significant contrast accurately reflects the true prevalence and distribution of individuals seeking help for weight loss in real life with and without BED. Fifth, the weekly medication adherence monitoring and the pill count at the end of the treatment may have been biased by the attempts of subjects, either intentionally or not, to misrepresent their compliance by discarding the prescribed medication rather than by consuming it. Despite these limitations, this study has some noteworthy strengths. To date, this is the first study that has evaluated the influence of *NUCB* polymorphism on the response to treatment with NB in patients with obesity and BED compared to patients with OBE without BED. This study is also the first to report an association between rs757081 *NUCB2* and the improvement in eating psychopathology in a cohort of patients after 16 weeks of treatment with NB. The results of this study could allow clinicians to identify a new population that may benefit from treatment with NB. For these reasons, other studies replicating and extending current results are needed.

### Practical Considerations

The results of this preliminary study could inform clinicians and researchers on the need to more accurately identify patients with OBE with/without BED that could benefit from treatment with NB. NB is a pharmacological treatment that has shown significant benefits for high-weight patients with not potentially life-threatening side effects, but a treatment response that may be influenced by genetics. Additionally, our results also suggest that FA may represent a peculiar phenotype of BED. Therefore, the presence of *NUCB2* polymorphism could be a valuable factor in determining the most effective treatment approach to enhance outcomes for individuals showing FA.

In the era of precision medicine, the results of the present study should serve as a driving force for the use of genetic tests in clinical practice, enabling the acquisition of an individual’s genetic profile to tailor treatment management. Indeed, this testing should not only assess their predisposition to being overweight, OBE, and associated comorbidities but also offer the potential to tailor personalized therapies.

## 5. Conclusions

Among the patients affected by BED undergoing NB treatment, a more favorable treatment response seems evident in individuals expressing the CG genotype of the *NUCB2* polymorphism. Taking into consideration all the limitations, these preliminary findings may imply that genetic investigations within the field of eating disorders could aid clinicians in selecting the most suitable treatment strategy, leveraging the polymorphic variations of susceptibility genes.

## Figures and Tables

**Table 1:** Sample description.

		Group 1 (Ob-BED) *N = 22*		Group 2 (Ob-noBED) *N = 20*			
		Mean	SD	Mean	SD	*t*	*p*
Age	(Years)	42	(12.5)	43.4	(14.7)	1.380	0.744
BMI	(kg/m^2^)	38.4	(6.7)	45.4	(10.4)	**2.617**	**0.012**
		**Fr**	**%**	**Fr**	**%**	***χ²***	***p***
Sex	Female	18	(81.8)	8	(40)	**6.010**	**0.013**
	Male	4	(18.2)	12	(60)		
Education	Elementary school	2	(9.1)	3	(15)	1.552	0.670
	Middle school I	5	(22.7)	5	(25)		
	High school II	11	(50)	12	(60)		
	University degree	4	(18.2)	0	(0)		
Employment	Unpaid activity	5	(22.7)	2	(10)	2.387	0.496
	Employed	13	(59.1)	11	(55)		
	Unemployed	3	(13.6)	6	(30)		
	Student	1	(4.5)	1	(5)		
Civil status	Single	9	(40.9)	7	(35)	0.155	0.925
	Married	11	(50)	11	(55)		
	Divorced	2	(9.1)	2	(10)		
rs757081 *NUCB2* genotype	CG	3	(13.6)	7	(35)	2.637	0.680
	CC	16	(72.7)	11	(55)		
	GG	3	(13.6)	2	(10)		

BED: *binge eating disorder*; BMI: *body mass index*; Fr: *frequency*; %: *percentage*; SD: *standard deviation*. Significant results are in bold.

## Data Availability

The data presented in this study are available upon reasonable request from the corresponding author.
